# Role of epigenetic factors in the selection of the alternative splicing isoforms of human *KRAS* in colorectal cancer cell lines

**DOI:** 10.18632/oncotarget.25016

**Published:** 2018-04-17

**Authors:** Ángela L. Riffo-Campos, Francisco Gimeno-Valiente, Fernanda M. Rodríguez, Andrés Cervantes, Gerardo López-Rodas, Luis Franco, Josefa Castillo

**Affiliations:** ^1^ Institute of Health Research INCLIVA, Valencia, Spain; ^2^ Present Address: Centro De Excelencia de Modelación Y Computación Científica, Universidad De La Frontera, Temuco, Chile; ^3^ Instituto De Ciencias Veterinarias Del Litoral (ICIVET Litoral), Universidad Nacional Del Litoral (UNL)/Consejo Nacional De Investigaciones Científicas Y Técnicas (CONICET), Santa Fe, Argentina; ^4^ Department of Medicine, Universitat De València, Valencia, Spain; ^5^ Centro De Investigación Biomédica En Red En Cáncer (CIBERONC), Madrid, Spain; ^6^ Department of Biochemistry and Molecular Biology, Universitat De València, Valencia, Spain

**Keywords:** epigenetics, chromatin structure, alternative splicing, colorectal cancer, KRAS isoforms

## Abstract

Mutation-driven activation of *KRAS* is crucial to cancer development. The human gene yields four mRNA splicing isoforms, 4A and 4B being translated to protein. Their different properties and oncogenic potential have been studied, but the mechanisms deciding the ratio 4A/4B are not known. To address this issue, the expression of the four *KRAS* isoforms was determined in 9 human colorectal cancer cell lines. HCT116 and SW48 were further selected because they present the highest difference in the ratio 4A/4B (twice as much in HCT116 than in SW48). Chromatin structure was analysed at the exon 4A, characteristic of isoform 4A, at its intronic borders and at the two flanking exons. The low nucleosome occupancy at exon 4A in both cell lines may result in a fast transcriptional rate, which would explain the general lower abundance of isoform 4A, also found in cells and tissues by other authors, but due to its similarity between both cell lines, chromatin structure does not influence alternative splicing. DNA methylation downstream exon 4A significantly differs in HCT116 and SW48 cells, but the CCCTC-binding factor, which affects the processivity of RNA polymerase and the alternative splicing, does not bind the differentially methylated sequences. Quantitative epigenetic analysis at mononucleosomal level revealed significant differences between both cell lines in H3K4me3, H3K27me3, H3K36me3, H3K9ac, H3K27ac and H4K20me1, and the inhibition of some histone-modifying enzymes alters the ratio 4A/4B. It can be concluded that the epigenetic modification of histones has an influence on the selection of isoforms 4A and 4B.

## INTRODUCTION

Human *KRAS* locus is located in chromosome 12 (25,204,789-25,250,936) and is transcribed from the reverse strand. Four mRNA isoforms, which result from alternative splicing, are reported in the Ensembl Genome database (accession number ENSG00000133703). Two of these mRNA isoforms are translated to protein, giving rise to the well-known KRAS-4A and KRAS-4B products. The two remaining mRNA isoforms contain open reading frames and may be putatively translated, but the actual existence of their protein products has not been reported to date. The first 164 residues of KRAS-4A and KRAS-4B are identical, but the C-terminal regions of the molecules (25 amino acids in isoform KRAS-4A and 24 in KRAS-4B), encoded by different exons, show a high variability. KRAS-4A and KRAS-4B are members of the Ras protein family, which also includes the highly homologous HRAS and NRAS. All of these proteins display GTPase activity and are involved in signalling pathways that regulate many cellular processes, including cell proliferation.

The functional cellular environment of the KRAS isoforms is the plasma membrane [1, 2], in which they are anchored through the farnesyl chains posttranslationally added at a C-terminal motif [3]. Isoform 4A is further directed to membrane by palmitoylation of a specific cysteinyl residue [4] and a sequence containing 7 lysines also contributes to the membrane localization of KRAS-4B through electrostatic interactions with the inner leaflet of the plasma membrane [2]. The function of the latter topogenic signal is regulated by the phosphorylation of serine 181, which is interspersed in the basic stretch [5]. KRAS-4A is directed to its final location via the Golgi system, while KRAS-4B goes from the endoplasmic reticulum to the plasma membrane through a different mechanism, which involves phosphodiesterase-δ [6, 7]. The structural bases for specific KRAS-4B/ phosphodiesterase-δ interaction have been recently studied [8].

The isoforms KRAS-4A and KRAS-4B behave in a different way in many other aspects. For instance, while the expression of *KRAS-4B* is ubiquitous in human tissues [9, 10], that of *KRAS-4A* is restricted to the gastrointestinal tract, kidney, lung and other tissues of endodermal origin [9] and similar results were obtained in mice [11, 12]. As to the oncogenic potential of both isoforms there is some discrepancy in the data recorded in the literature. It is clear that the ratio of both isoforms is altered in cancer [9] and, while the focus-inducing potential of KRAS-4B is lower than that of KRAS-4A, the latter isoform do not induce cell migration and KRAS-4B does [13]. These early results are in agreement with those of King *et al.*, who found that knockdown of KRAS-4A reduces proliferation of renal cell carcinoma [10]. However, after the results obtained in mice, KRAS-4A is classically considered to be proapototic, whereas KRAS-4B is antiapoptotic [14].

At any rate, it is clear that changes in the ratio of splicing isoforms 4A and 4B exist when comparing their expression levels in different cell lines or tissues, but the results reported in the literature are also controversial. It has been described that the ratio *KRAS-4A*/*KRAS-4B* is significantly reduced in 6 CRC cell lines when compared with cells derived from normal colon and in tumour from 4/9 patients with sporadic CRC when compared with the adjacent normal mucosa [9] and similar results were obtained in adenomas of mouse small intestine in the absence of *KRAS* -activating mutations [15]. In agreement with these results, Chung *et al.*, by transfecting mouse livers with different *KRAS* constructs, found that the survival of mice carrying mutation-activated *KRAS-4A* is significantly better than that of animals transfected with mutated *KRAS-4B* [16] and Luo *et al.* propose that the exon specifically included in *KRAS-4A* confers the gene a certain tumour-suppressor function [17]. The above data suggest that mutation-activated *KRAS4B* is more oncogenic than activated *KRAS4A*, and yet there are some discordant views in the literature. For instance, Wang *et al.* reported that increase of the ratio *KRAS-4A*/*KRAS-4B* in murine lung correlated with higher susceptibility to tumour development [12] and a major role in mouse lung carcinogenesis has been ascribed to *KRAS-4A* [18]. It has also been described that the expression of this isoform in human colorectal cancer and adenomas is higher than in the adjacent normal tissues [19].

Whatever the causes of the above discrepancies, it seems clear that it is worth studying the factors that decide the proportion of *KRAS* isoforms. Differential splicing is affected by many factors, including chromatin structure and epigenetic modifications near the splicing sites (for reviews, see [20, 21]). None of these factors have been studied in the human *KRAS* locus, although there is evidence that *cis*-acting elements, presumably located in introns or in 3’-untranslated regions decide the balance between *KRAS-4A* and *KRAS-4B* in murine lung cancer [22]. These circumstances prompted us to investigate the distribution of *KRAS* isoforms, including the non-coding ones, which might possess a regulatory role, in human CRC cell lines and to determine the chromatin structure and its epigenetic modifications in the regions involved in the selection among the different isoforms.

## RESULTS

### Expression of *KRAS* isoforms in different cell lines

**Figure 1:**
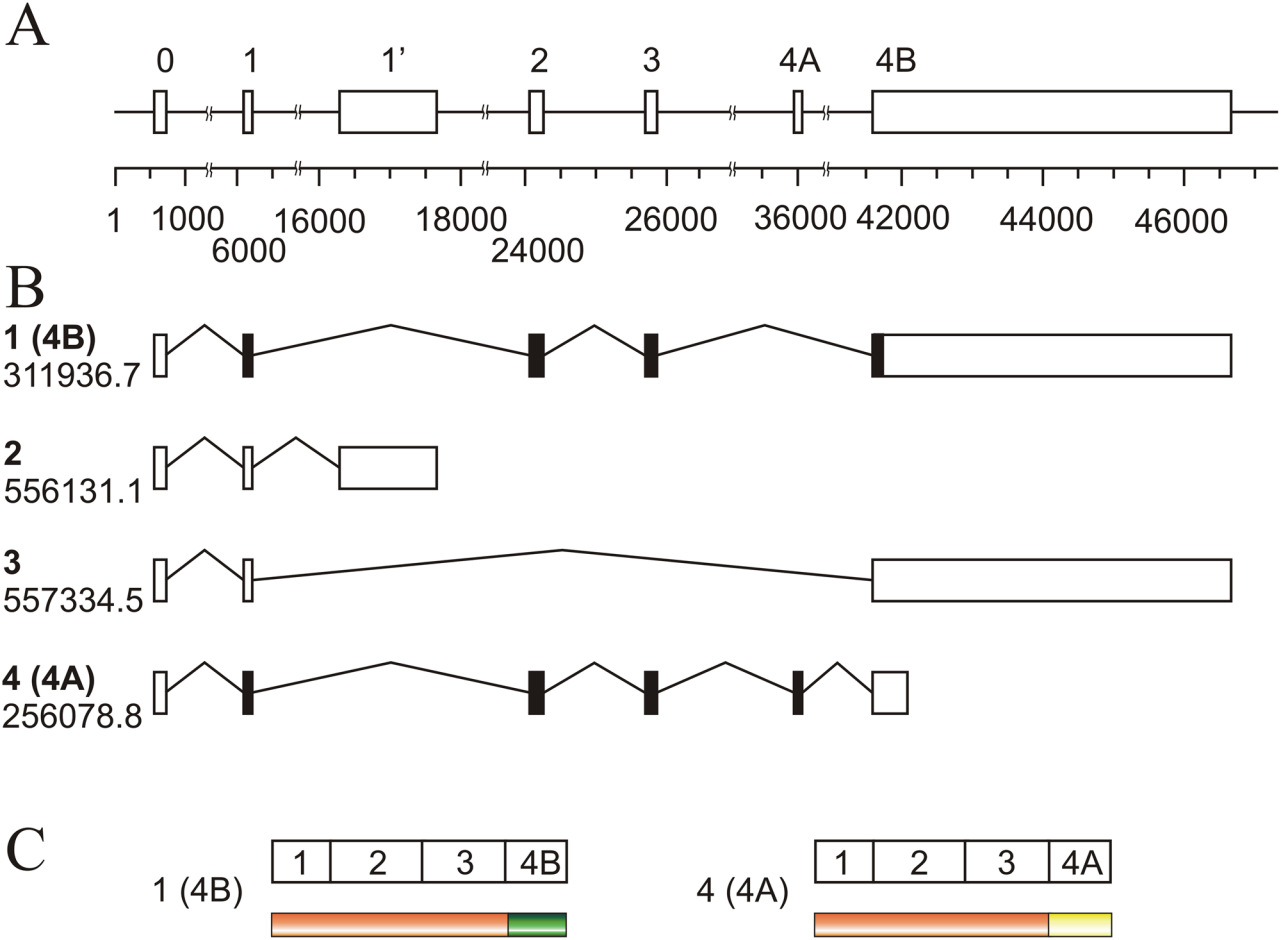
Schematic representation of the human *KRAS* locus **(A)** map of the entire locus in which the exons are depicted as boxes and identified in the widespread manner, which only numbers the translatable exons (1 to 4B). The 5’ non-translatable exon present in all mRNA isoforms is designed as exon 0, and the non-translatable exon downstream of exon 1, is numbered as 1’. The numbers below the scale give, in base pairs, the absolute position within the locus. **(B)** mRNAisoforms resulting from alternative splicing, identified with the number given in the text (bold lettering) and with the Ensembl Genome database numbering; black boxes indicate the translatable exons. **(C)** map of the two known protein products of the gene, showing the common (orange) and the variable sequences.

To facilitate the interpretation of the results, a map of the human *KRAS* locus is given in Figure 1. The four possible transcripts are identified by the isoform number and code included in the ENSEMBL database. The conventional exon numbering, which correspond only to the translated ones (see, for instance, ref. [23]), is used. The untranslated exon present upstream of exon 1 in all the isoforms is further referred to as exon 0 and the exon downstream exon 1, characteristic of isoform 2, is designed as 1’.

**Figure 2:**
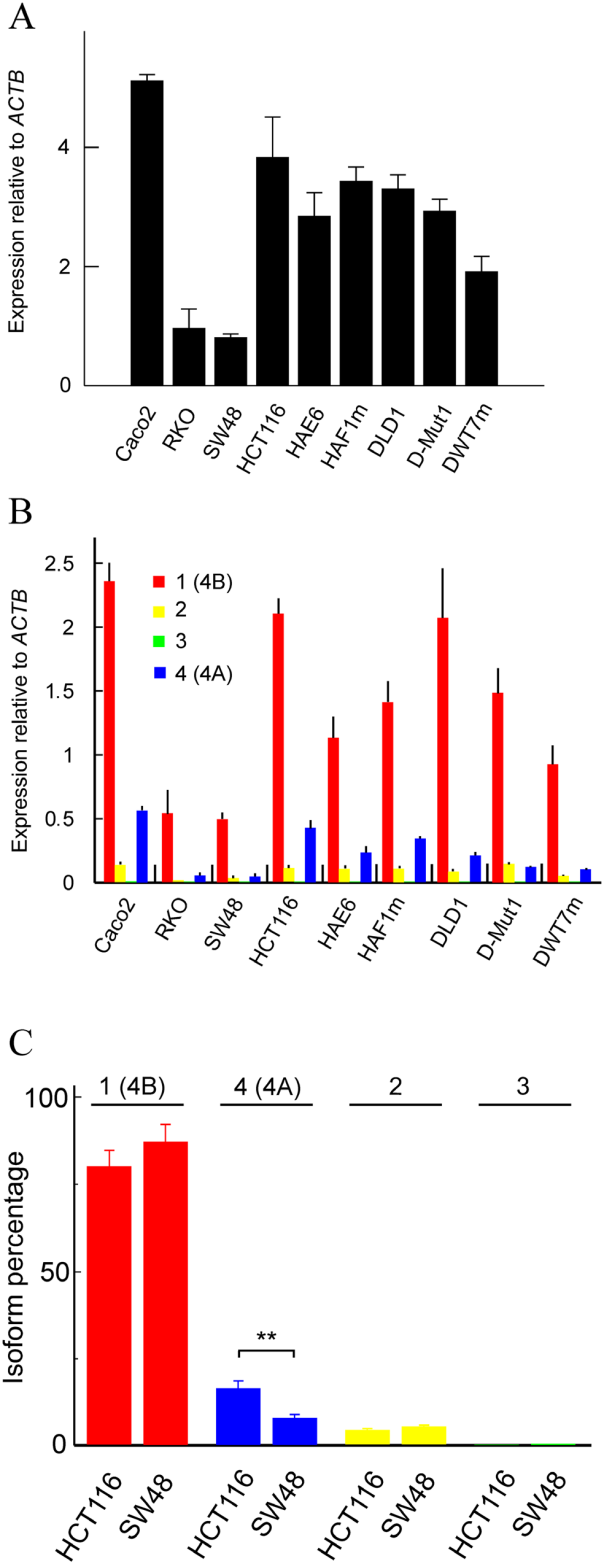
Transcription level of the whole *KRAS* gene and of its isoforms in several human CRC cell lines **(A)** expression, relative to the *ACTB* gene, of the whole gene in 9 cell lines. **(B)** expression, relative to the *ACTB* gene, of the four isoforms in those cell lines. **(C)** percent expression of the four isoforms in HCT116 and SW48 cells. The results were obtained by RT-qPCR in triplicate and expressed as the mean ± standard deviation.

We first quantified the transcription of whole *KRAS* in 9 CRC cell lines (Figure 2A) using primers from exon 0, common to all mRNA isoforms. The relative abundance of each four transcripts was then determined (Figure 2B). To differentiate among the four isoforms we designed the primers given in Supplementary Table 1. Isoform 1 (4B) was analysed with a forward primer from exon 3 and a reverse primer spanning exons 3 and 4B. Isoform 2 was quantified by using a forward primer from exon 1 and a reverse primer from exon 1’, which is unique to this isoform. To analyse isoform 3, a forward primer from exon 1 and a reverse one spanning exons 1 and 4B were used. Finally, isoform 4 (4A) was quantified with a forward primer from exon 4A, which is present only in this isoform, and a reverse primer from 5’ end of exon 4B. The location of those primers is depicted in Supplementary Figure 1. To correct for the possible differences in the efficiency of the primers, we determined the efficiency factors, which are also given in Supplementary Table 1. The slight differences in efficiency did not affect the results of the RT-qPCR experiments given in Figure 2B.

The expression of whole *KRAS* is highly variable from line to line, being higher in Caco2 and lower in RKO and SW48. The expression level is roughly similar in lines HCT116 and DLD1 and the cells derived from these parental lines show a lower expression level, although a slight compensatory effect for the loss of one of the alleles seems to occur (Figure 2A). The expression of the individual isoforms, relative to *ACTB*, is given in Figure 2B. Isoform 1 (4B) is the most abundant in all the cell lines studied; present results agree with those of Tsai *et al.* [23]. The level of isoform 3 is negligible in all the cell lines, but the expression of the non-translated isoform 2 is comparable, or even slightly higher than that of isoform 4 (4A).

In view of the above mentioned interest of studying the mechanisms deciding the relative abundance of the different splicing isoforms of *KRAS*, especially the isoforms 1 (4B) and 4 (4A), we selected the cell lines SW48 and HCT116. This selection was based in the fact that they are the lines showing a greater difference in the ratio between both isoforms. This is especially apparent in Figure 2C, in which the percentage of the four isoforms is given. The percentage of isoform 4 (4A) in HCT116 doubles that in SW48 at the expense of isoform 1 (4B), while the proportion of isoform 2 is roughly similar in both cell lines and that of isoform 3 is negligible. In other words, exon 6 (4A) is skipped in SW48 cells twice more than in HCT116 cells.

#### Chromatin structure at the differential splicing sites of HCT116 and SW48 cell lines

**Figure 3:**
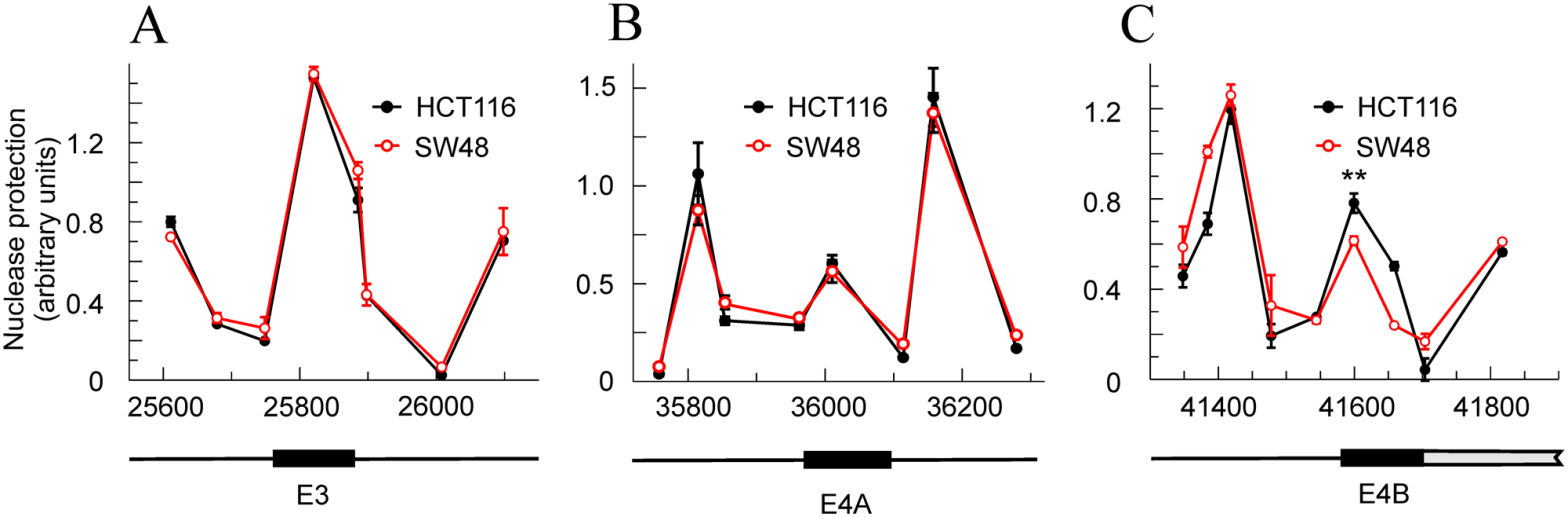
Nucleosome occupancy at the exons involved in alternative splicing in HCT116 and SW48 cells **(A)** exon 3. **(B)** exon 4A. **(C)** exon 4B. The plots give the nuclease protection in arbitrary units against the position of each amplicon centre in the locus. The maps below identify the exon location as black rectangles. In (C), the gray rectangle refers to the non-translatable, transcribed exon. The results correspond to three determinations for each amplicon (Panels A and C) or to six determinations from two independent experiments (Panel B) and are given as the mean ± standard deviation. ^**^ p<0.01.

Taking into account that the chromatin structure is a factor influencing the splicing events [20], the nucleosomal organization of the *KRAS* locus in exons 3 and 4A and in the translatable 5’ end of exon 4B was studied. These regions include the adjacent splicing sites and other potential sequences that may influence inclusion or skipping of exon 4A. The micrococcal nuclease protection data, obtained at the amplicons defined in Supplementary Table 2, are compatible with the occupancy of the three exons by nucleosomes. The size of the exons is, respectively, 165, 124 and 117 bp, so there is room in them for a single nucleosome, and the width of the protected areas are also compatible with the presence of a single nucleosome over each exon (Figure 3A-3C). The sequence-based prediction retrieved from the NuPop program is compatible with the presence of nucleosomes over exons 3 and 4A, but not with the presence of a nucleosome in the 5’ end of exon 4B, in which the probability of assembling a nucleosome is very low, in accordance with the high content of A and T in that region. In spite of these circumstances, the results suggest that the nuclease protection observed in the 5’ end of exon 4B (Figure 3C) is actually due to the presence of a nucleosome. As it will be shown later, the Nuc-ChIP experiments gave positive results at the amplicon centred at position 41600 and a quantitative determination of the concentration of H3 by using a Nuc-ChIP assay with an antibody against the C-terminal end of the histone gave at least twice as much H3 over the exon 4B than over the exon 4A. Thus, it is highly probable that the nuclease protection at the 5’ end of exon 4B is due to the presence of a *bona fide* nucleosome.

The profiles of micrococcal nuclease protection are almost identical in HCT116 and SW48 cell lines, and only a slight difference is observed in the nucleosome occupancy over exon 4B. Within the resolution margin of this assay, the flanks of the exons, which contain the 3’ and 5’ splicing sites, as well as the polypyrimidine tract immediately upstream of the 5’ sites, seem to be unprotected in both cell lines and, then, accessible to the spliceosome assembly. In view of the above results, it seems that the chromatin structure has no influence on the differences in exon 4A skipping between both cell lines.

#### DNA methylation downstream of exon 4A

**Figure 4:**
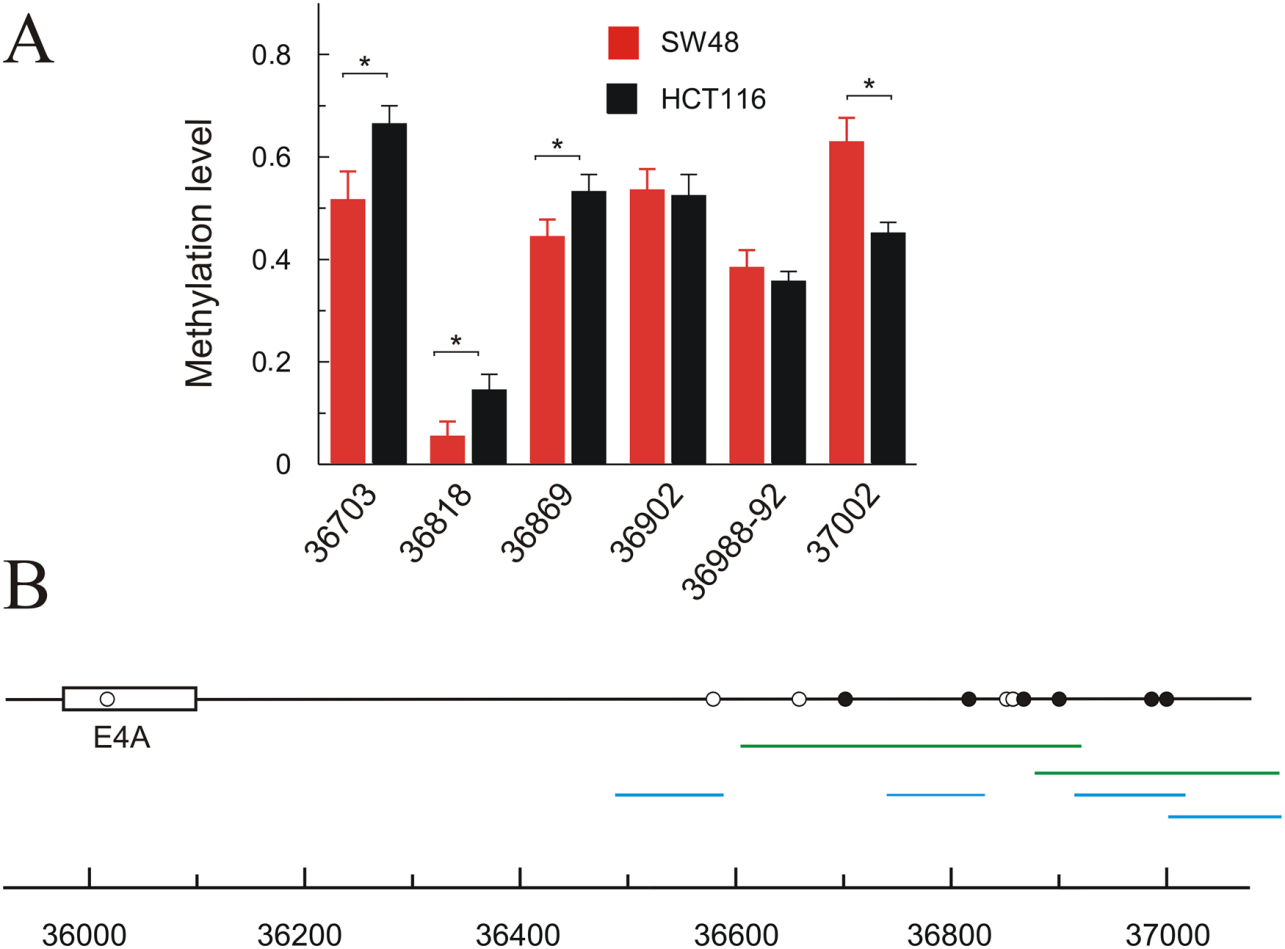
Methylation level of CpGs downstream exon 6 (4A) in HCT116 and SW48 cell lines **(A)** the level of methylation in a quantitative scale (average of 5-10 determinations) is plotted against the position of the analysed CpGs in the locus; the CpGs showing a significant difference (p<0.05) between both cell lines are identified. **(B)** map showing the position of the CpGs; filled circles indicate those analysed for their methylation level, while empty circles refer to those CpGs, which could not be analysed by the method used. The amplicons used to evaluate CpG methylation are shown in green, and those used for qPCR analysis of CTCF binding are represented in blue.

DNA methylation can also affect exon skipping in several ways [24]. To check whether distinct methylation levels may be the cause of the differential level of *KRAS* isoforms 4 (4A) and 1 (4B) in HCT116 and SW48, we analysed DNA methylation downstream of exon 4A. As shown in Figure 4, there is a single CpG dinucleotide within exon 4A and this low CpG content may be related to its low nucleosome occupancy (see Figure 3B) [25]. Several CpG sites are clustered in the downstream intron starting approximately at 500 bp 3’ to exon 4A, but only 6 out of these sites could be analysed to quantify their methylation degree following the used procedure (Figure 4B). Subtle, but significant, differences between SW48 and HCT116 cell lines were observed in the three CpG sites analysed between 36700 and 36870, which showed a more intense methylation in HCT116 (Figure 4A).

The possibility that these differences in methylation resulted in a distinct CCCTC-binding factor (CTCF) recruitment was next studied, as it has been described that the presence of bound CTCF downstream of exons causes a diminution of the elongation rate of RNA pol II, which in turn may affect the inclusion of the exon due to kinetic factors [24]. ChIP analysis, followed by qPCR evaluation of amplified sequences, revealed that CTCF does not appreciably bind the region studied. Actually, the concentration of DNA sequences immunoprecipitated with an anti-CTCF antibody is similar to that recovered without added antibody (Supplementary Table 3).

#### Epigenetic modification of histones in the regions involved in alternative splicing

Taking into account that splicing usually is a co-transcriptional event, it is known that the presence of some epigenetic marks in nucleosomes surrounding the splicing sites may influence exon skipping and/or inclusion [20, 21]. As the differences in the selection between the 4A and 4B *KRAS* isoforms in HCT116 and SW48 cell lines is not probably determined by chromatin structure or CTCF binding, the differential presence of epigenetic marks in the nucleosomes covering exons 3, 4A and 4B was next studied by the Nuc-ChIP procedure. The following histone post-translational modifications (PTMs) were analysed: H3K4me3, H3K9me3, H3K27me3, H3K36me3, H3K9ac, H3K27ac and H4K20me1. These histone PTMs rank among the marks most frequently associated with splicing events (see [26] and references therein). Two independent Nuc-ChIP experiments were carried out at the amplicons corresponding to the three nucleosomes positioned on exons 3, 4A and 4B. The PCR quantifications were done in triplicate in both experiments. With only an exception (see below), the differences in the studied epigenetic marks between HCT116 and SW48 cells were not significant in exons 3 and 4A, while most of them were significant in the nucleosome covering exon 4B.

**Figure 5:**
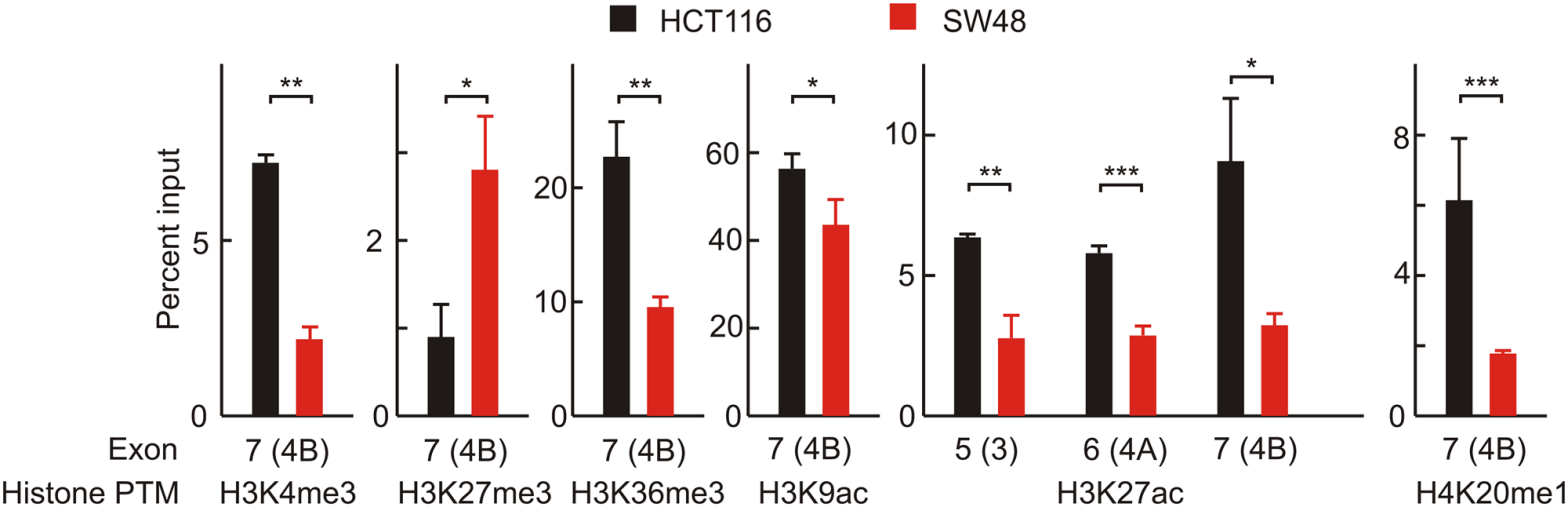
Nuc-ChIP analysis of histone epigenetic modifications in nucleosomes located over the exons 3, 4A and 4B Only those histone PTMs that significantly differ between HCT116 and SW48 cells are depicted. Three independent Nuc-ChIP experiments were carried out and the figure gives the results of one representative experiment. qPCR analysis were carried in triplicate and the mean ± standard deviation is plotted. ^*^ p<0.05; ^**^ p<0.01; ^***^ p<0.001.

Only the results giving significant differences between HCT116 and SW48 cell lines are depicted in Figure 5. The comparison of the results with the marks on H3K27 provides an internal control of the validity of the experiments, because the trends of acetylation and methylation run in the opposite direction, as expected for two mutually exclusive PTMs.

**Figure 6:**
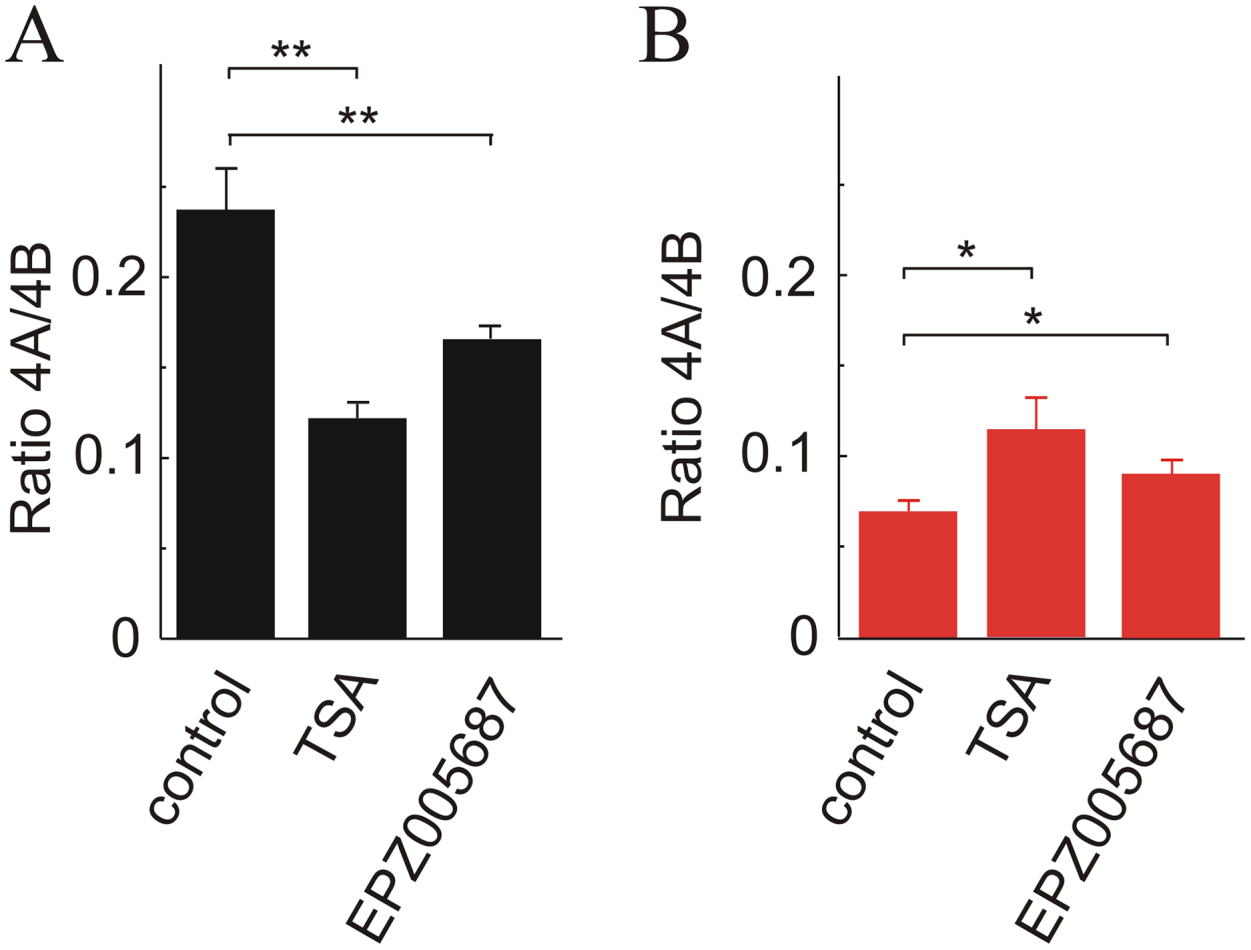
Effects of the changes in the level of H3 acetylation and H3K27 methylation on the *KRAS* 4A/4B ratio TSA was used to inhibit histone deacetylases and EPZ005687 to inhibit EZH histone methyltransferases. **(A)** HCT116 cell line. **(B)** SW48 cell line.

H3K27ac is the only epigenetic mark which shows significant differences in the three nucleosomes studied, being more intense in the three exons in HCT116 cells. These cells are also more heavily marked in the nucleosome covering exon 4B in four out of the other histone PTMs that show significant differences, namely H3K4me3, H3K36me3, H3K9ac and H4K20me1. Only H3K27me3 is more intense in SW48 than in HCT116 (Figure 5). To check whether differences in histone epigenetic modifications are actually related to alternative splicing, the ratio of isoforms 4A/4B was studied after inhibiting histone deacetylases with trichostatin A (TSA), and EZH1/2 histone methyltransferases with EPZ005687. Inhibition of the latter enzymes was selected because they specifically catalyse the methylation of H3K27. As the other methylatable lysines studied in the experiment of Figure 5 are modified by several redundant methyltransferases [27], inhibition of all of them results in cell lethality. As expected, treatment with TSA increases the acetylation level of bulk H3 and EPZ005687 reduces the methylation of H3K27 (Supplementary Figure 2). Figure 6 shows that reducing the level of H3K27me3 causes a significant increase in the 4A/4B ratio in the SW48 cell line. In both cell lines, the TSA-induced change of H3 acetylation leads to significant changes in the 4A/4B ratio, which resulted levelled after the treatment.

## DISCUSSION

The results described in the present paper add some details to our knowledge on the distribution of *KRAS* isoforms and on the mechanisms involved in their selection. First, in spite of the differences in the expression of the whole gene observed among the different cell lines analysed (Figure 2A), isoform 1 (4B) is the more abundant in every case (Figure 2B). With a few exceptions, this is a common feature in both, cell lines and human normal and cancerous tissues [9, 17, 23]. The splicing variants 2 and 3 have received little or no attention in the literature. The fact that the translation of their mRNAs had not been demonstrated to date may be a cause for that lack of interest.

As far as we know, present paper describes for the first time a study of the distribution of those isoforms in several CRC cell lines. The presence of isoform 3 is negligible in all the cell lines studied, but isoform 2, which includes exon 1’ and a premature transcription termination signal (Figure 1), is clearly detectable. It is known that a non-translatable mRNA splice variant of *HRAS*, which includes the otherwise skipped exon 5, plays a regulatory role in the expression of the gene [28], and it would be interesting to know whether *KRAS* isoform 2 performs a similar role.

The differences in the ratio between isoforms 1 (4B) and 4 (4A) among the different cell lines found in the present paper, as well as the differences observed by many authors, put forward the question as to what splicing regulatory mechanisms decide between the inclusion or skipping of exon 4A in the mature transcript. In fact, exons 4A and 4B cannot be regarded as mutually exclusive exons at the level of mature mRNA, because the 5’ end of exon 4B is present in the mature transcript of both isoforms 1 (4B) and 4 (4A). In the latter isoform, exon 4B is not translated, because the included exon 4A contains a stop codon (TAA) in the 3’ end.

Intron and exon definition may obey to two different mechanisms, occurring either at the level of the mRNA precursor, or at the DNA level [21]. Taking into account that in most cases splicing is a cotranscriptional event, the second mechanism seems to be the most common one. In this case, chromatin structure plays a fundamental role. Nucleosomes are usually positioned in exons and their presence causes RNA polymerase to reduce its processivity [29], helping the splicing factors to be recruited to the nascent pre-mRNA [21].

To investigate the mechanisms deciding the inclusion of exon 4A, a comparison of the chromatin structure of the HCT116 and SW48 cells was first done. Of note, although the differences between both cell lines are negligible, the nucleosome occupancy is lower at exon 4A than at the other exons. It is also much lower than in the proximal regions of the flanking introns (Figure 3B), so the processivity of RNA polymerase II is not reduced at its passage through exon 4A. Interestingly, the presumably fast processivity of RNA polymerase II through exon 4A may explain why the isoform containing this exon is less abundant than isoform 1 (4B) in all the cell lines studied in the present paper as well as in the patient samples and cell lines analysed by other authors [23].

The epigenetic differences between HCT116 and SW48 cell lines in the regions of interest to the present study were also examined. It is known that CpG methylation of DNA and histone PTMs can affect splicing through the “recruitment coupling” model [20]. For instance, DNA methylation at CpG dinucleotides prevents the binding of CTCF at overlapping target sites of the factor. As CTCF represents an obstacle to RNA polymerase II processivity, CTCF binding at non-methylated CpG containing sites favours the inclusion of weak upstream exons [30]. Nevertheless, the present results (Figure 4 and Supplementary Table 3) allowed us to conclude that CTCF-dependent kinetic factors do not represent a main mechanism to decide the inclusion of exon 4A.

Finally, the histone PTMs were analysed by Nuc-ChIP in the three nucleosomes positioned over the exons 3, 4A and 4B. We have previously used this technique [31, 32] and its advantages have been recently reviewed [33]. Most of the significant differences in histone PTMs between HCT116 and SW48 cell lines are found in the nucleosome positioned in exon 4B (Figure 5).

The experiment of Figure 6 supports the hypothesis that changes in histone PTMs result in an alteration of the alternative splicing events. The enhancement of the acetylation of H3 is associated with a significant increase in the 4A/4B ratio in SW48 cell line (Figure 6B), in which the low level of H3 acetylation (Figure 5) is linked to a low 4A/4B ratio. SW48 cells are characterized by a high level of H3K27me3 (Figure 5) and a lower 4A/4B ratio and, therefore, a diminution of methylation might well be linked to an increase of the isoform ratio. The effects of inhibiting EZH1/2 histone methyltransferases on HCT116 cells are not so easy to explain. Anyway, the results of Figure 6 clearly shows that the histone PTMs examined are in some way responsible for the discrimination between *KRAS* 4A and 4B isoforms.

It is not an easy task to ascribe the differences in the isoform ratio to a given epigenetic mark. In spite of the efforts that have been carried out in the last years to find a splicing code (for an early proposal, see [34]), an unambiguous correlation between histone epigenetic marks and splicing events does not exist. Many of the studies in that sense have been carried out by means of genomic approaches [35] and, while the information provided by these methods is highly valuable from a statistical point of view, particular genes may not obey to those general rules.

The differences in histone PTMs between HCT116 and SW48 cells shown in Figure 5 are mainly confined to the nucleosome over the exon 4B. The question arouses as to whether epigenetic differences in exon 4B may cause, in some way, the observed differential skipping of exon 4A. The recruitment model [36–38] provides a plausible hypothesis to explain the influence of epigenetic modifications of the nucleosome over exon 4B on the skipping of the upstream exon. This model assumes that some histone PTMs recruit, through an adaptor, a splicing regulator that, in turn, binds a target regulatory site in nascent pre-mRNA. The present fine analyses at mononucleosomal resolution may be relevant to deepen the knowledge of the splicing epigenetic code and of the relationships between histone modifications and cancer progression. In this way, novel candidate therapeutic targets might be eventually found, by using either epigenetic modulators [39], or drugs targeted to the splicing machinery [40].

## MATERIALS AND METHODS

### Cell lines

The human CRC cell line HCT116 (ATCC CCL-247) and their derivatives HAE6 (a gift from Dr B. Vogelstein) and HAF1m [41], DLD1 (ATCC CCL-221) and their derivatives D-Mut1 (a gift from Dr B. Vogelstein) and DWT7m [42] were grown in McCoy's 5A medium (Sigma, St. Louis, MO, USA). The cell lines RKO (Horizon Discovery, Cambridge, UK) and Caco-2 (ATCC HTB-37) were grown in Dulbecco's modified Eagle's medium (DMEM) and the cell line SW48 (Horizon), was grown in RPMI 1640 medium (Sigma). All media were supplemented with 10% heat inactivated foetal bovine serum, 1% penicillin-streptomycin and 1% L-glutamine (Sigma), and the cultures were maintained under standard conditions. Table 1 shows the relevant genotypes and sources of the cell lines. The cell lines obtained from other authors were not further authenticated.

**Table 1:** Cell lines used in this paper

Cell line	Relevant genotype				Source
	*KRAS*	*BRAF*	*PIK3CA*	*TP53*	
**DLD1** D-Mut1 DWT7m	G13D/wt G13D/- G12D^*^/-	wt	E545K; D549N	S241F	ATCC CCL-221 Dr Vogelstein Riffo-Campos *et al.* [24]
**HCT116** HAE6 HAF1m	G13D/wt G13D/- A146T/-	wt	H1047R	wt	ATCC CCL-247 Johns Hopkins University Roda *et al.* [23]
**RKO**	wt	V600E	H1047R	wt	Horizon Discovery
**SW48**	wt	wt	wt	wt	Horizon Discovery
**Caco2**	wt	wt	wt	E204X	ATCC HTB-37

The parental cell lines are represented in bold characters and, when appropriate, the mutant variant is listed below them. The mutation marked by an asterisk affects only 20% of cell population.

To inhibit histone modifying enzymes, cells were loaded in 6-well plates and treated with either 0.25 μM TSA (Sigma) or 20 μM EPZ005678 (Selleckchem) for 24 h.

### Determination of transcript levels

RNA extraction, retrotranscription to cDNA and qPCR were carried out as previously described [42], using the β-actin gene (*ACTB*) as standard. The primers used for whole *KRAS* and for the different transcripts resulting from alternative splicing are given in Supplementary Table 1. The isoforms are identified by their number in the ENSEMBL database, followed in the case of isoforms 1 and 4, by the more widespread nomenclature 4B and 4A in brackets.

To determine the efficiency of each qPCR primer set, a standard curve was prepared. The Ct values for serial template dilutions of cDNA are plotted against the logarithm of the dilution factor and the calculated slope gives a measure of the efficiency factor, which was used for normalization to compare the results obtained at different amplicons.

### Analysis of chromatin structure

Nucleosome occupancy was determined by the micrococcal nuclease protection assay [32], using tiled amplicons of about 100 bp in size. The primers used are given in Supplementary Table 2. The experimental results were compared with the output of the sequence-based prediction of positioning carried out using the NuPoP software tool [43].

### Analysis of DNA methylation

Quantitative DNA methylation analysis was carried out essentially as described by Coolen *et al.*[44]. Briefly, the method uses a T7-promoter-tagged PCR amplification of bisulphite-modified DNA, followed by generation of a single-stranded RNA molecule and subsequent base-specific cleavage (3′ to either rUTP or rCTP) by RNase A. The mixture of cleavage products, which differ in length and mass, are analysed by MALDI-TOF-MS. Changes in nucleotide sequence after bisulphite treatment, which reflects the differences in the methylation profile of original DNA, give origin to different fragment masses in the assay. The abundance of each fragment (signal/noise level in the spectrum) is indicative of the amount of DNA methylation in the analysed sequence.

Previously, bisulphite treatment of genomic DNA (1 μg) was carried out with the EZ-96 DNA methylation kit (Zymo Research), following the manufacturer’s protocol. For quantitative methylation analysis an AGENA’s MassARRAY platform was used. PCR primers for the amplification of the different regions of the *KRAS* locus downstream of exon 4A were designed by using *Epidesigner* (AGENA) and their sequences and location were given, respectively, in Supplementary Table 4 and Figure 4B.

The PCRs were carried out in a 5 μl format with 10 ng/μl bisulfite-treated DNA, 0.2 units of *Taq*DNA polymerase (AGENA), 1 × *Taq* buffer, and 200 nM PCR primers. Dephosphorylation of unincorporated dNTPs was performed as recommended by the manufacturer. The reaction mixtures were further diluted with 20 μl of H_2_O and conditioned with 6 mg of CLEAN Resin (AGENA) for optimal mass-spectra analysis.

### Chromatin immunoprecipitation (ChIP)

To investigate the binding of CCCTC-binding factor (CTCF) to chromatin, ChIP analysis was carried out after sonicating the chromatin to an average fragment size of 250-300 bp following the previously described procedure [45, 46]. The sequences of primers used for qPCR were given in Supplementary Table 5 and their location is depicted in Figure 4B. Although the amplicons are not tiled, the average size of chromatin fragments ensured that CTCF is absent from the entire region under consideration. Epigenetic modifications of histones were studied at nucleosomal level by Nuc-ChIP [31, 32]. The following antibodies were used: anti-H3K9ac (Abcam, ab-4441); anti-H3K9me3 (Abcam, ab-8898); anti-H3K27ac (Abcam, ab-4729); anti-H3K27me3 (Millipore, 07-449); anti-H3K4me3 (Abcam, ab-8580); anti-H3K36me3 (Abcam, ab-9050); anti-H3K20m (Abcam, ab-9051); anti-β-actin (Abcam, ab-8227).

### Statistical analysis

Quantitative values are expressed as mean ± SD. Data in the different PCR determinations were compared by two-tailed t-test. Differences were considered significant at p<0.05.

## SUPPLEMENTARY MATERIALS FIGURES AND TABLES


