# Supplementary material for: Role of epigenetic factors in the selection of the alternative splicing isoforms of human KRAS in colorectal cancer cell lines
Source: Oncotarget. 2018 Apr 17;9(29):20578–89. doi: 10.18632/oncotarget.25016 (PMC5945503; doi:10.18632/oncotarget.25016)
Supplement: Supplementary file 1 [file oncotarget-09-20578-s001.pdf]

Role of epigenetic factors in the selection of the alternative splicing isoforms of human *KRAS* in colorectal cancer cell lines

SUPPLEMENTARY MATERIALS

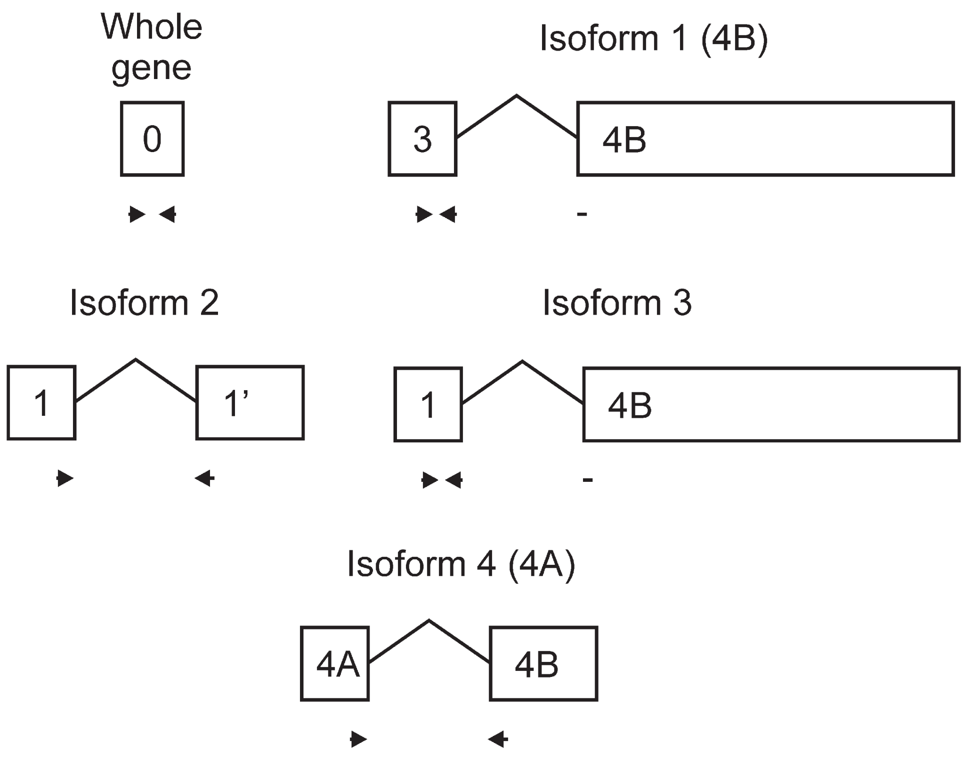

Supplementary Figure 1: Location of the primers used for qPCR analysis of the transcription of whole *KRAS* gene and of its individual isoforms. The boxes stand for the exons, identified by their numbers (see Figure 1). The primers are depicted as arrows below the exons. A broken arrow means that the sequence of the primer corresponds to the junction between the two exons.

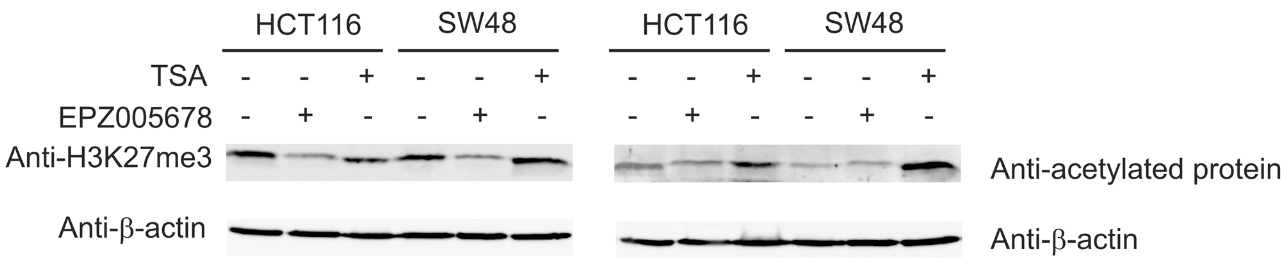

Supplementary Figure 2: Western blots showing the effect of the use of inhibitors on the level of H3K27me3 and of H3 acetylation. The inhibitors added to the HCT116 and SW48 cell cultures and the primary antibodies used were indicated. β-actin was used as loading control.

Supplementary Table 1: Primers used for PCR determination of transcript levels

Isoform	Primers		Size	Efficiency
	Forward	Reverse		
Whole	CTTGTGGTAGTTGGAGCTGGT	TGTTGGATCATATTCGTCCACAA	90	1.76
1 (4B)	CACAAAACAGGCTCAGGACTT	ATCAACACCCTGTCTTGTCTT	106	1.77
2	AGTGCCTTGACGATACAGCTAAT	AAGGCATCATCAACACCCTCTATT	80	1.75
3	ACAATTTCAAGAGGAAATCCCCG	TCTGCCAATTCCATGAGATGCT	92	1.80
4 (4A)	AAGAAAAGACTCCTGGCTGTGT	AGGCATCATCAACACCCAGAT	70	1.75

The location of these primers is depicted in supplementary Figure 1.

Supplementary Table 2: Primers used for determination of nucleosome occupancy and Nuc-ChIP

Amplicon	Primers		Size
	Forward	Reverse	
25606	TGAGAGAGATACAAGGTTTCTGTT	TCCACTACACCAAATTTTCCTTCC	84
25675	TTTGGTGTAGTGGAAACTAGGAA	TCTTTCAAAACCTGTCCACAACT	82
25746	AGTTGTGGACAGGTTTTGAAAG	ACATCTTCAGAGTCCTTAACTCTT	106
25819	GGACTCTGAAGATGTACCTATGGTC	TGTGTCTACTGTTCTAGAAGGCAA	70
25885	ACACAAAACAGGCTCAGGACT	TCTTGTCTTTGCTGATGTTTCAA	71
25898	GCTCAGGACTTAGCAAGAAGT	TCAGTGTTACTTACCTGTCTTGT	77
26010	CTGTTTTCTGCAAAATCATAACTGT	CAATGCCCTCTCAAGAGACAA	120
26102	GTCTCTTGAGAGGGCATTGCT	ACATGTGCTCAGAATTGAAGAGA	103
35757	ACCTGTGTTTAGGCTTGTCAT	TGGGCTAGAATCCTGGTTTGTT	113
35814	ACTGACTTTTAAGAACAAACCAGGA	TGTTTGAATAAAACTGAGGATGCAG	70
35853	ACAAACCAGGATTCTAGCCCA	GCCATGTGCAAGAAGTTTGAGA	120
35961	CTTGCACATGGCTTTCCCAG	TTGTCGGATCTCTCTCACCA	120
36009	TGTGTTTTACAATGCAGAGAGTG	AGGAGTCTTTTCTTCTTTGCTGA	101
36112	AGACTCCTGGCTGTGTGAAAAT	GTGGTTGCCACCTTGTTACCT	120
36156	TAATTTTGGCAGAAAGCAGATGTC	ACACCTAAGTAGTTCTAAAGTGGTT	70
41350	GACTTAGGTTTGCCAATGTGGA	AGGATAACCAATGGCACAGAA	97
41386	GTTCTCCTGTGAAAAAGTCG	ACAGGAACACTAATTTTCAC	84
41420	CTGTGCCATTGGTTATCCTTGT	CATCCGCATAGGTGTTTTGTCA	78
41479	ACAAAAACCTATGCGGATG	TCTCTTCAGGCAACTGAA	79
41545	TTCAGTTGCCTGAAGAGA	TAGCTTCATGTGTACAGG	90
41600	CCTGTACACATGAAGCCATCGT	AAGGCATCATCAACACCCTGA	75
41659	TCAGGGTGTTGATGATGCCT	ACCATCTTTGCTCATCTTTTCTTT	76
41704	GAAAAGATGAGCAAAGATGGT	CACACTTTGTCTTTGACTTCT	55
41817	GGCATACTAGTACAAGTGG	TAACAGTCTGCATGGAGCA	99
ACTB	GTGCTATCCCTGTACGCCTC	GAGGGCATACCCCTCGTAGA	99

Supplementary Table 3: Results of the ChIP analysis for quantitative determination of CTCF binding

Amplicon	HCT116		SW48	
	**anti-CTCF**	**No antibody**	**anti-CTCF**	**No antibody**
36538	0.0047±0.0026	0.0051±0.0037	0.0064±0.0037	0.0043±0.0024
36785	0.0079±0.0042	0.0061±0.0053	0.0049±0.0038	0.0149±0.0092
36965	0.0021±0.0017	0.0030±0.0022	0.0051±0.0022	0.0016±0.0010
37053	0.0034±0.0025	0.0073±0.0015	0.0068±0.0018	0.0036±0.0009

The table gives the results of qPCR analysis of DNA recovered from immunoprecipitated chromatin, either with an anti-CTCF antibody or without antibody added.

Supplementary Table 4: Primers used for determination of DNA methylation

Primers		Size
**Forward**	**Reverse**	
TTAGTGAGAAATTTGGGAATTTTAGG	AACTCCTAACCTCAAATAATCACCC	308
TTTTAGTATTTTGGGAGGTTTAGG	TCAAACTAAAATACAATAATACATTCTCAA	231

The last column gives the size of the amplicons defined by these primers; their location is depicted in Figure 4B.

Supplementary Table 5: Primers used for CTCF ChIP analysis

Amplicon	Primers		Size
	Forward	Reverse	
36538	ATTGAATGGCCCAGAAGGTGG	AGATGGCGAACTTAGGCAGAA	100
36785	GGCCCCTATCATTTCTTGTTGG	AGTGTATCTCTCCGGTTTCACA	91
36965	TCACCTGAGGTCAGGAGTTCA	TAGCTGGGATTACAGGCACGT	103
37053	ACGTGCCTGTAATCCCAGCTA	AGCTGGAGTGCAATGGTGCAT	105

The location of the amplicons is shown in Figure 4B.
